# Efficient Pseudotyping of Different Retroviral Vectors Using a Novel, Codon-Optimized Gene for Chimeric GALV Envelope

**DOI:** 10.3390/v13081471

**Published:** 2021-07-27

**Authors:** Manuela Mirow, Lea Isabell Schwarze, Boris Fehse, Kristoffer Riecken

**Affiliations:** 1 Research Department Cell and Gene Therapy, Department of Stem Cell Transplantation, University Medical Center Hamburg-Eppendorf (UKE), 20246 Hamburg, Germany; m.mirow@uke.de (M.M.); l.schwarze@uke.de (L.I.S.); 2 German Center for Infection Research (DZIF), Partner Site Hamburg-Lübeck-Borstel-Riems, 20246 Hamburg, Germany

**Keywords:** retroviral vectors, adoptive immunotherapy, chimeric antigen receptor (CAR), T-cell receptor (TCR), gene therapy, pseudotyping, GMP

## Abstract

The Gibbon Ape Leukemia Virus envelope protein (GALV-Env) mediates efficient transduction of human cells, particularly primary B and T lymphocytes, and is therefore of great interest in gene therapy. Using internal domains from murine leukemia viruses (MLV), chimeric GALV-Env proteins such as GALV-C_4070A_ were derived, which allow pseudotyping of lentiviral vectors. In order to improve expression efficiency and vector titers, we developed a codon-optimized (co) variant of GALV-C_4070A_ (coGALV-Env). We found that coGALV-Env mediated efficient pseudotyping not only of γ-retroviral and lentiviral vectors, but also α-retroviral vectors. The obtained titers on HEK293T cells were equal to those with the classical GALV-Env, whereas the required plasmid amounts for transient vector production were significantly lower, namely, 20 ng coGALV-Env plasmid per 10^6^ 293T producer cells. Importantly, coGALV-Env-pseudotyped γ- and α-retroviral, as well as lentiviral vectors, mediated efficient transduction of primary human T cells. We propose that the novel chimeric coGALV-Env gene will be very useful for the efficient production of high-titer vector preparations, e.g., to equip human T cells with novel specificities using transgenic TCRs or CARs. The considerably lower amount of plasmid needed might also result in a significant cost advantage for good manufacturing practice (GMP) vector production based on transient transfection.

## 1. Introduction

Integration into the host cells’ genome is an obligatory step in the life cycle of retroviruses. Based thereon, vectors derived from γ-retro- as well as (later) lenti- and α-retroviruses have become important tools for gene-therapy applications that require permanent expression in replicating cell populations, such as hematopoietic stem cells or T lymphocytes [1,2,3].

The cell-specificity of retroviruses and vectors derived thereof is largely determined by their envelope (Env) proteins that mediate binding to cellular surface proteins also referred to as “viral receptors” (e.g., CD4 for HIV-Env). Early on in retrovirology, it was shown that the natural Env protein of retroviral particles could be replaced by suitable equivalents (glycoproteins) derived from other viruses [3,4,5,6]. Such exchanges have been subsumed under the term pseudotyping and today include not only the use of naturally occurring, but also engineered viral envelopes [7]. Depending on the vector, exchange of the original Env has several potential advantages, e.g., broader or narrower range of target cells, lower toxicity, and higher stability allowing enrichment of vector particles by ultracentrifugation [6]. Therefore, pseudotyping, particularly of lentiviral vectors, e.g., with the VSV-G protein, has become an indispensable tool to ensure efficient vector production and achieve high-level gene transfer to desired cell populations [7]. On the other hand, the VSV-G protein has certain limitations, not the least its relatively high cytotoxicity, impeding its stable expression in permanent producer cell lines. Therefore, the pseudotyping potential of Env proteins derived from other retroviruses has been investigated, including those derived from murine retroviruses (MuLV10A1) [8], non-human primate viruses (Gibbon Ape Leukemia Virus (GALV) [4,9], Baboon retroviral envelope (BaEV) glycoprotein [10], and Feline Leukemia Virus (RD114) [5].

Already by the 1990s, GALV-Env-pseudotyped γ-retroviral vectors were shown to mediate efficient transduction of human primary T cells even with non-concentrated γ-retroviral vectors stocks [9,11]. In contrast, pseudotyping of lentiviral vectors with original GALV-Env was inefficient. This limitation was overcome by fusing the external, cell-binding subunit of GALV-Env to the transmembrane C-terminus of the amphotropic MLV 4070A thus generating the chimeric (GALV-C_4070A_) envelope [12,13]. Indeed, we previously applied lentiviral vectors pseudotyped with the chimeric GALV-C_4070A_ Env for efficient transduction of primary T and B lymphocytes [14,15].

The recent breakthroughs in adoptive immunotherapy with gene-modified T cells expressing chimeric antigen receptors (CARs) [16] have further highlighted the need for methods that efficiently transduce large numbers of T cells in accordance with good manufacturing practice (GMP) regulations. Currently, manufacture of most viral CAR vectors is based on transient transfection of so-called producer cell lines with separate packaging plasmids encoding all necessary viral proteins as well as the vector’s genome. While sufficiently effective, this process is relatively variable and expensive. The need for large amounts of highly purified plasmids is one of the factors contributing to these high costs [17].

The ambiguity of the genetic code has resulted in the variable use of the 61 individual codons by different organisms. Indeed, a codon preferably used to encode a defined amino acid in a given organism might rarely be present in another. This observation has led to the concept of codon optimization [18] which represents a method that uses synonymous codon changes to increase protein production in defined target cells. The approach is based on the assumptions that rare codons are limiting the translation rate, and that changing synonymous codons does not affect the structure and function of a protein. While viruses generally adapt to the preferred codon usage of their host organisms during evolution, this adaptation is often hampered by their own biology. For example, the small genome of retroviruses requires the use of interlaced open-reading frames (ORFs) and the overlap of ORFs with indispensable structural elements both limiting the usage of perfectly adapted codons in the virus genome. In addition, the use of non-optimal codons might reflect another layer of regulation to ensure correct stoichiometry of the different viral proteins, which in case of retroviruses are all expressed by a single promoter. However, separation of the necessary genes on individual packaging plasmids has opened the possibility to codon-optimize cDNAs for improved vector packaging [18,19]. In contrast, codon optimization has been less frequently used to codon-optimize Env proteins. A recent paper even reported functional impairment of the RD114TR envelope glycoprotein after “codon optimization” due to incorrect protein processing [20].

Here, we describe the development and testing of a codon-optimized (co) gene variant of the chimeric GALV-C4070A-env. The new coGALV-Env was used to pseudotype lentiviral as well as γ- and α-retroviral vectors. We investigated the impact of codon optimization on vector titers and transduction efficiencies in cultured cell lines (HEK293, K562) and in primary human T cells. Our data demonstrate substantially improved translation efficiency with coGALV-env supporting its use for the production of all three vector types (lentiviral, γ- and α-retroviral vectors).

## 2. Materials and Methods

### 2.1. Plasmids and Codon Optimization

The plasmid phCMV-GALV-C4070A encoding the chimeric GALV-C4070A-env [14] was a kind gift from Carol Stocking. The corresponding codon-optimized gene version coGALV-env was designed with GeneOptimizer software and synthesized by a contract manufacturer (Thermo Fisher Scientific, Waltham, MA, USA). A comparison of the complete original and the optimized sequences is provided in Supplementary Figure S1; vector maps are depicted in Supplementary Figure S2. Large amounts of plasmids were generated in *E. coli* TOP10 using standard recombinant-DNA techniques (QIAquick Spin Maxiprep Kit, QIAGEN, Hilden, Germany). The new plasmid containing the codon-optimized coGALV-env has been deposited at Addgene (#172217).

### 2.2. Generation of Viral Particles

The used lentiviral vector LeGO-mOrange2 (Addgene, #85212) was described previously [21]. It was produced following our established protocol [22], termed “standard scale”, in 10-cm cell culture dishes (a detailed protocol is available online at http://www.LentiGO-Vectors.de; assessed 25 July 2021). HEK293T cells (CRL-11268), obtained from the American Type Culture Collection (ATCC; Manassas, VA, USA), were used for LV production and titration. For determination of the optimal amount of env-plasmid, virus production was conducted on a small scale, deviating from the standard protocol as follows: 8 × 10^5^ 293T cells were seeded per well of a 6-well plate in 1 mL DMEM medium. Consequently, 1/6 of the plasmid amounts were used for the DNA/CaCl_2_ mix. Instead of adding DNA/CaCl_2_ to HBS dropwise while air was blown into the HBS-buffer, the DNA/CaCl_2_ was mixed with the HBS buffer immediately in small-scale production. Viral particles were harvested after 12, 24, and 36 h. Harvest was performed by removing cellular debris via filtration (0.45 µm), and the viral supernatants were stored at −80 °C.

The γ-retroviral vector pRSF91.GFP.pre* [23] as well as both α-retroviral vectors (pAlpha.SIN(noTATA).EFS.EGFP.wPRE and pAlpha.SIN(noTATA).MPSV.EGFP.wPRE [24]) were kindly provided by Axel Schambach (MHH Hannover, Germany).

### 2.3. Titration of Vector Supernatants on 293T Cells

5 × 10^4^ 293T cells in 500 µL DMEM medium were seeded in a 24-well plate and incubated at 37 °C for 2–5 h until the cells were attached. Polybrene (Sigma-Aldrich, St. Louis, MO, USA) was added at a concentration of 8 µg/mL to each well, before 0.5 to 100 µL of the viral supernatant were given to the cells. Afterwards, the plates were centrifuged for 1 h at 25 °C and 1000× *g* (spin-inoculation). After 3 days of incubation at 37 °C, transduction efficiencies were analyzed by flow cytometry.

### 2.4. Transduction of Myeloid K562 Cells and Primary Human T Lymphocytes

Peripheral blood mononuclear cells (PBMCs) isolated from buffy coats (from healthy blood donors, kindly provided by the Institute of Transfusion Medicine, UKE, after informed consent) were stimulated with CD3/CD28 beads and IL-2 to selectively expand T cells. To this end, frozen PBMCs were thawed, washed, taken up in TexMACS medium with autologous serum, and subsequently seeded into a 6-well plate with 6 × 10^6^ cells in 4 mL per well. Further, 200 U/mL IL-2 (Proleukin 100,000 U/mL, Novartis, Basel, Schweiz) and CD3/CD28 beads (T Cell TransAct, Miltenyi Biotec, Bergisch Gladbach, Germany) at a 1:100 ratio were added [11,25]. After an incubation time of 3 days, 2 × 10^5^ primary T cells per well in 300 µL TexMACS medium with autologous serum were seeded in a 24-well plate, where only the 8 inner wells of the plate were used (in our experience, the outer wells sometimes were not even, leading to cells being pelleted at the border of a well after centrifugation, potentially interfering with transduction efficiencies). A total of 8 µg/mL of polybrene was added to each well, before 5 µL to 100 µL of the viral-particle containing supernatant were given to the cells. Subsequently, the plates were centrifuged for 1 h at 25 °C and 1000× *g*. After 3 days of incubation at 37 °C, the cells were measured by flow cytometry.

Transduction of K562 cells (ATCC: CCL-243) was tested using 5 × 10^4^ cells in 500 µL RPMI medium per well in a 24-well plate, where only the 8 inner wells of the plate were used. A total of 8 µg/mL of polybrene was added to each well before 1 µL to 50 µL of the vector supernatants were given to the cells. Afterwards, the plates were centrifuged, incubated, and analyzed in the same way as the primary T cells.

### 2.5. Statistical Analysis

If not indicated otherwise, the data shown represent means and standard deviations of three independent experiments. Statistical significance was determined using Student’s t-test.

## 3. Results

### 3.1. Impact of Codon Optimization on Lentiviral Vector Titers

With the aim of improving the expression strength of the envelope plasmid phCMV-GALV-C4070A encoding the chimeric GALV-C4070Aenv [14], a codon-optimized version coGALV-env was designed. A comparison of the complete original and the optimized sequences is provided in Supplementary Figure S1; plasmid maps are depicted in Supplementary Figure S2.

We first assessed a potential influence of codon optimization on the titer of GALV-Env-pseudotyped lentiviral vectors (LVVs). To do so, we produced LeGO-mOrange2 vectors in 293T cells following our standard protocol (see Material and Methods), where the VSV-G protein was replaced by GALV-Env. Based on our own experience and data published by others [12,13,26,27], between 400 and 1000 ng GALV-env plasmid is required per 1 Mio cells to reproducibly ensure high-titer LVV production. In line with that, in preliminary experiments that were aimed at defining the optimal concentration range for the two plasmids, we determined peak titers (up to >4 × 10^6^/mL) with approximately 550 ng GALV-env plasmid, whereas low amounts (≤100 ng/Mio cells) in some cases resulted in titers substantially below 10^5^/mL. In contrast, for coGALV-Env peak titers of >3 × 10^6^/mL could be obtained with approximately 10 ng plasmid per 1 Mio cells in this screening (Supplementary Figure S3).

**Figure 1:**
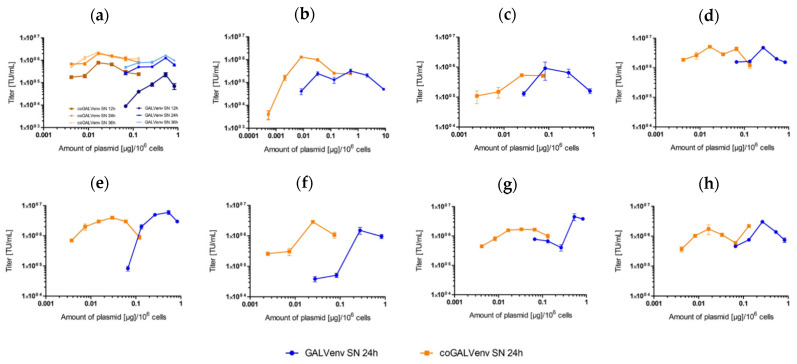
Comparative titration of lentiviral LeGO-mOrange2 vectors produced with standard vs. codon-optimized (co) GALV-env. (**a**) Much lower amounts of coGALV-env as compared to GALV-env were required to obtain maximal titers. Supernatants were harvested 12, 24, and 36 h after transfection of 293T cells. As evident, maximal titers were in all cases obtained with >20 times lower amounts of coGALV-env as compared to GALV-env; (**b**–**h**) titration experiments for seven additional, independent vector productions (only the 24 h data point is shown). Means and standard deviations of triplicates are shown. Small error bars are part hidden behind data point symbols.

In the following, we tested different amounts of plasmids encoding either non-codon-optimized or coGALV-env in parallel experiments with identical passages of producer cells. In order to facilitate direct comparison, we focused on the respective optimal concentration ranges determined for the two different plasmids in the preliminary experiments. Supernatants were collected after approximately 12, 24, and 36 h; titers were assessed on 293T cells based on mOrange2 expression by flow cytometry. We obtained very similar results for all different time points (Figure 1a). Therefore, here, we will focus on the 24 h data for the sake of clarity. The results of eight independent vector productions (all performed in triplicates for each concentration indicated) are displayed in Figure 1.

**Figure 2:**
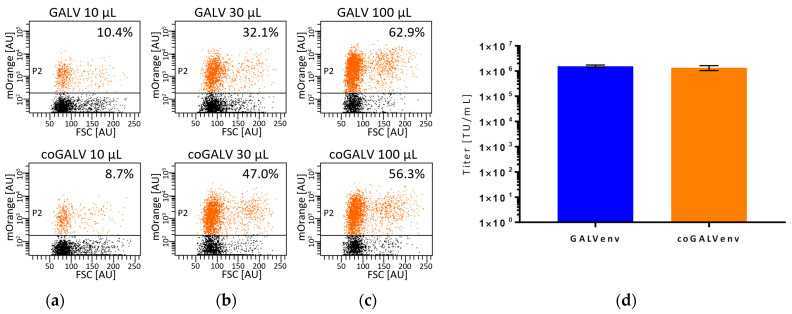
Titers of lentiviral LeGO-mOrange2 vectors produced in standard-scale with GALV-env and coGALV-env. GALV-env-vectors were produced in triplicates with 640 ng of plasmid/10^6^ cells, coGALVenv-vectors with 20 ng of plasmid/10^6^ cells. (**a**–**c**) Exemplary dotplots of transduced primary human T cells for three different amounts (10, 30, 100 µL) of vector-containing supernatant produced with GALV-env or coGALV-env. Clear-cut separation of transduced vs. non-transduced cells even in demanding cells facilitates unequivocal determination of transduction rates indicated; (**d**) Means and standard deviations of titer determination performed in triplicates. No significant difference in titers as measured on 293T cells was seen by t-test. FSC = forward scatter, AU = arbitrary units of area signal.

Based on the data shown in Figure 1, we determined the optimal plasmid amounts for lentiviral vector production to be 640 ng/10^6^ 293T cells for standard GALV-env and 20 ng/10^6^ 293T cells for coGALV-env. Next, we directly compared these amounts of plasmids in a standard-scale vector production (in triplicates). As shown in Figure 2, we indeed obtained identical titers with the >30 times lower amount of plasmid encoding the coGALV-Env.

### 3.2. Titer of GALV-Env vs. coGALV-Env Pseudotyped γ-Retroviral (γ-RVVs) and α-Retroviral Vectors (α-RVVs)

γ-retroviral vector systems are frequently used in gene therapy, particularly for T-cell transduction. In contrast, α-retroviral vector systems have not yet been broadly employed for stable clinical gene transfer. However, based on their favorable integration profile and the possibility to establish permanent producer cell lines, α-RVVs have been suggested as a very promising alternative to LVVs and γ-RVVs [24,25,28]. In the next part of the project, we thus addressed the possibility of using the coGALV-Env to pseudotype both γ-RVVs and α-RVVs.

**Figure 3:**
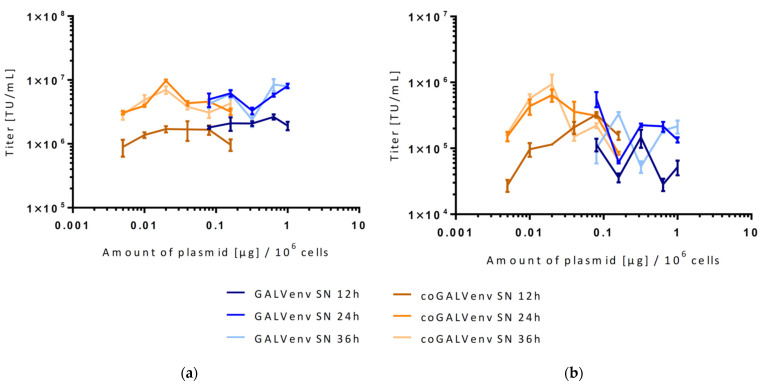
Titers of (**a**) γ- and (**b**) α-retroviral vectors produced with GALV-env or coGALV-env as determined on 293T cells. For transient vector production, the following plasmid amounts per 10^6^ producer cells were used: GALV-env: 1 μg, 0.64 μg, 0.32 μg, 0.16 μg and 0.08 μg, coGALV-env: 0.16 μg, 0.08 μg, 40 ng, 20 ng, 10 ng, and 5 ng of plasmid. Data points shown represent means of triplicates. SN = supernatant, TU = transducing units.

To determine whether codon optimization has the same effect on the production of γ-RVVs and α-RVVs as on LVVs, a new vector preparation was conducted. Generally, based on preliminary results and published data on optimal plasmid concentrations [24,29] we used the same experimental setup for vector preparation as shown for LVVs, i.e., multiple parallel vector productions with increasing amounts of GALV-env and coGALV-env and three times for harvest of supernatants. During vector production, it was interesting to note that the 293T cells producing α-RVVs were less attached to the surface of the tissue culture dish than those cells producing γ-RVVs. Unexpectedly, for both γ-RVVs and α-RVVs, vector productions with standard GALV-env did not reveal distinct optima of plasmid amounts, but titration curves showed rather wave-like shapes. Nevertheless, vector titers were in the expected range for both vector types (10^7^ transducing units [TU]/mL for γ-RVVs and 10^6^ TU/mL for α-RVVs), independent of the used env plasmid (GALV-env or coGALV-env). Most importantly, for both vector systems, maximal titers were again obtained with lower (approximately 8–10 times) plasmid amounts of coGALV-env (20 ng of plasmid/10^6^ 293T cells) as compared to standard GALV-env (Figure 3).

In order to address the potential impact of the internal promotor on vector titer, the original EFS promotor in the α-RV was replaced by a stronger MPSV promotor, and a new vector production was performed for both γ-RVVs and α-RVVs following the same scheme as in Figure 3. Interestingly, very similar titration curves with no clear titer maxima were found for both vector types (not shown). These data might indicate that relatively broad ranges of env plasmid amounts around the determined maxima still facilitate efficient vector production.

**Figure 4:**
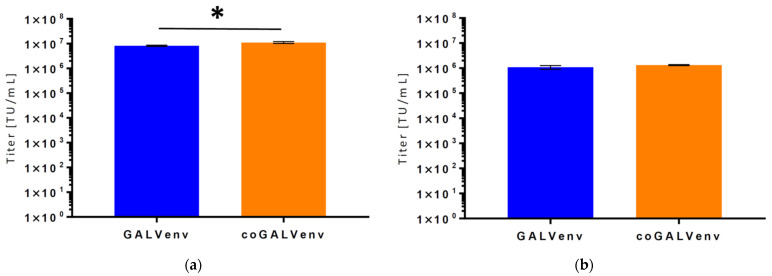
Titers of vectors produced in standard-scale with GALV-env and coGALV-env for γ-RVs and α-RVs. GALV-env-vectors were produced with 160 ng of plasmid/10^6^ cells, coGALV-env-vectors with 20 ng of plasmid/10^6^ cells. Titers of (**a**) γ-RVVs and (**b**) α-RVVs were determined on 293T cells. Data shown are the mean of triplicates. TU = transducing units, * *p* < 0.01 (*t*-test).

Based on these data, we used the following plasmid amounts (per 10^6^ producer cells) for standard-scale manufacture of both vector types (γ-RVVs and α-RVVs)—GALV-env: 160 ng, coGALV-env: 20 ng. The supernatants produced in the standard-scale runs for the two vectors were titrated on 293T cells as above; results are displayed in Figure 4. As evident, we again obtained very similar titers for both vector constructs, despite the fact that we used eight times lower amounts of the plasmid encoding coGALV-Env.

### 3.3. Transduction of K562 Cells and Primary Human T Lymphocytes with All Three Vector Types

In the final set of experiments, we used all three vector types to transduce hematopoietic cells, namely, the human erythro-leukemia cell line K562, and, more importantly, primary human T lymphocytes. K562 cells were chosen as a broadly used model cell line, whereas primary T cells represent a highly relevant target for genetic modifications in immunotherapy. Moreover, all three vector types have been successfully used to transduce primary T cells previously.

**Figure 5:**
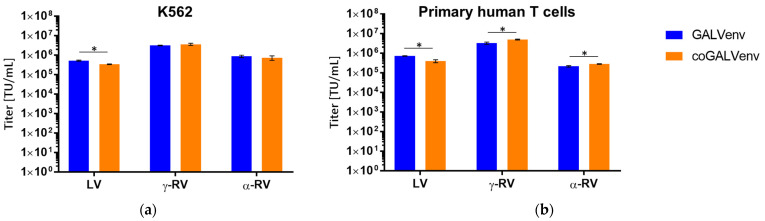
Titers of standard-scale produced supernatants of the three vector types (for LVVs, γ-RVVs, and α-RVVs) on K562 cells and primary human T lymphocytes. LVVs were produced with 640 ng and γ-RVV as well as α-RVV with 160 ng GALV-env plasmids per 10^6^ producer cells. In contrast, for production of all coGALV-env vectors, only 20 ng of plasmid/10^6^ cells was used. Titers were determined using dilution series on (**a**) K562 and (**b**) primary T lymphocytes. Shown are means of triplicates. TU = transducing units, * *p* < 0.01 (*t*-test).

In order to directly assess the potential impact of the GALV-env plasmid (standard vs. codon-optimized), we determined titers of the vector preparations from the standard-scale productions for all three vector types (LVVs, γ-RVVs, and α-RVVs). To this end, we used standard dilution series as described above. Cell-type specific titers of the different vector preparations are depicted in Figure 5, separately for K562 (Figure 5a) and T cells (Figure 5b). As evident, only minor differences between the two envelopes without clear tendency in favor of one or the other were found for the three vector types. In particular, in primary human T cells, we observed slightly (factor 1.8) higher titers for LVV pseudotyped with the standard GALV-Env, while for both γ-RVVs and α-RVVs the titers were slightly higher with the coGALV-Env (by factors 1.5 and 1.3, respectively). Although these minor variances between GALV-env and coGALV-env would not be expected to be relevant in most cases, our data confirmed the notion that in the direct comparison of the three vectors, GALV-env pseudotyped γ-RVVs might be most useful for primary human T cells.

## 4. Discussion

Based on their ability to transduce human hematopoietic cells with minimal cytotoxicity, GALV-Env-pseudotyped retroviral vectors are of great interest for various gene-therapy applications. Indeed, already in the 1990s it was shown for γ-RVVs that the GALV-Env protein mediates efficient transduction of primary T lymphocytes [9,11]. Subsequently, the GALV-Env protein was modified to allow pseudotyping of LVVs [12,13,14,15]. More recently, pseudotyping of α-RVVs was also achieved but resulted in comparatively low vector titers [23,24].

In view of the recent progress in adoptive immunotherapy with T cells expressing tumor-targeting TCRs [30] and, particularly, CARs [16], efficient gene transfer into T lymphocytes has become a very urgent task. Here, we asked whether codon-optimization of the GALV-env ORF might contribute to improved vector titers (in particular for α-RVVs) and the reduction of plasmid amounts required for production of all vector types. The latter point will be relevant in the context of manufacturing schemes based on transient transfection of producer cell lines where large amounts of plasmid of good manufacturing practice (GMP) quality are required. Notably, manufacturing costs of cell-therapy drugs still represent an important limitation for broader application [31].

For γ-RVVs and LVVs, we observed that application of the coGALV-Env during transient vector production resulted in very similar titers as the original plasmid. However, the amount of necessary GALV-env plasmid could be reduced by a factor of >30 (20 ng instead of 640 ng per 1 Mio producer cells) for LVVs and by a factor of eight for γ-RVVs (20 ng instead of 160 ng/10^6^ cells). Notably, the applied quantity of just 20 ng of packaging plasmid for coGALV-env is substantially lower than the amounts used by many other groups to pseudotype γ-RVVs and LVVs [12,13,25,26,28]. We also obtained relatively good titers (>10^6^ transducing units per ml of non-concentrated supernatant) for α-RVVs, which is somewhat in contrast to previous work [23,24]. Again, the necessary amount of plasmid was strongly reduced for the codon-optimized construct (20 ng vs. 160 ng per 1 Mio producer cells) and >10 times lower than previously reported [23].

Codon optimization was previously used to optimize production of pseudotyped LVVs and γ-RVVs, e.g., with Spike protein from SARS-CoV-1 [32], parainfluenza type 3 envelope proteins [33], and a truncated HIV-Env [34]. In striking contrast, Zucchelli et al. observed impaired functionality of codon-optimized RD114-TR, another envelope protein derived from a γ-retrovirus that is commonly used for pseudotyping [20]. The latter data have thus proven that codon optimization of the env gene does not necessarily improve protein expression or vector titers.

In our work we did not see any functional impairment for any of the three tested vector types (LVVs, γ-RVVs, and α-RVVs) produced with the coGALV-env. In direct comparison, coGALV-Env pseudotyped γ-RVVs showed the highest titers (approximately 1 × 10^7^ TU per mL on 293T cells). This was to be expected, since GALV-Env is derived from a γ-RVV and had to be modified (chimeric GALV-Env) to be efficiently incorporated into the other vector particles [12,13,23]. With regard to practical use, the observed high transduction rates on primary T lymphocytes with coGALV-Env γ-RVVs might be of particular interest.

## 5. Conclusions

The coGALV-Env facilitates efficient pseudotyping of lentiviral, γ-retroviral, and α-retroviral vectors. For all three vector systems, significantly lower amounts of plasmid of coGALV-env are required to gain identical titers and transduction rates, as compared with classical GALV-env. These data might be of particular relevance for large-scale, GMP vector production using transient producer cell lines.

## Supplementary Materials

The following SM is available online at https://www.mdpi.com/article/10.3390/v13081471/s1, Figure S1: Comparison of the complete original and the optimized sequences of GALV-env. Figure S2: Vector maps of both envelope expressing plasmids used in this work. Figure S3: Dose-finding of coGALV-env plasmid amount needed for efficient packaging of lentiviral vectors.
