# Supplementary material for: Efficient Pseudotyping of Different Retroviral Vectors Using a Novel, Codon-Optimized Gene for Chimeric GALV Envelope
Source: Viruses. 2021 Jul 27;13(8):1471. doi: 10.3390/v13081471 (PMC8402753; doi:10.3390/v13081471)
Supplement: Supplementary file 1 [file viruses-13-01471-s001.zip › viruses-1268938-supplementary.pdf]

Figure S1: Comparison of the complete original and the optimized sequences of GALVenv.

```
                   10        20        30        40        50        60        70        80
           ....|....|....|....|....|....|....|....|....|....|....|....|....|....|....|....|
GALVenv    ATGGTATTGCTGCCTGGGTCCATGCTTCTCACCTCAAACCTGCACCACCTTCGGCACCAGATGAGTCCTGGGAGCTGGAA
coGALVenv  ATGGTTTTGCTGCCTGGCTCTATGCTGCTGACCAGCAACCTGCACCACCTGAGACACCAGATGAGCCCTGGCAGCTGGAA

                   90       100       110       120       130       140       150       160
           ....|....|....|....|....|....|....|....|....|....|....|....|....|....|....|....|
GALVenv    AAGACTGATCATCCTCTTAAGCTGCGTATTCGGCGGCGGCGGGACGAGTCTGCAAAATAAGAACCCCCACCAGCCCATGA
coGALVenv  GCGGCTGATCATCCTGCTGAGCTGTGTGTTTGGCGGCGGAGGCACAAGCCTGCAGAACAAGAATCCCCACCAGCCTATGA

                  170       180       190       200       210       220       230       240
           ....|....|....|....|....|....|....|....|....|....|....|....|....|....|....|....|
GALVenv    CCCTCACTTGGCAGGTACTGTCCCAAACTGGAGACGTTGTCTGGGATACAAAGGCAGTCCAGCCCCCTTGGACTTGGTGG
coGALVenv  CACTGACCTGGCAGGTCCTGTCTCAGACCGGGGATGTCGTGTGGGATACAAAGGCTGTGCAGCCTCCTTGGACCTGGTGG

                  250       260       270       280       290       300       310       320
           ....|....|....|....|....|....|....|....|....|....|....|....|....|....|....|....|
GALVenv    CCCACACTTAAACCTGATGTATGTGCCTTGGCGGCTAGTCTTGAGTCCTGGGATATCCCGGGAACCGATGTCTCGTCCTC
coGALVenv  CCTACACTGAAGCCTGATGTGTGTGCCCTGGCCGCCTCTCTGGAATCTTGGGATATTCCTGGCACCGACGTGTCCAGCAG

                  330       340       350       360       370       380       390       400
           ....|....|....|....|....|....|....|....|....|....|....|....|....|....|....|....|
GALVenv    TAAACGAGTCAGACCTCCGGACTCAGACTATACTGCCGCTTATAAGCAAATCACCTGGGGAGCCATAGGGTGCAGCTACC
coGALVenv  CAAGAGAGTCAGACCTCCTGACAGCGATTACACCGCCGCCTACAAGCAGATCACATGGGGCGCCATCGGCTGCAGCTACC

                  410       420       430       440       450       460       470       480
           ....|....|....|....|....|....|....|....|....|....|....|....|....|....|....|....|
GALVenv    CTCGGGCTAGGACTAGAATGGCAAGCTCTACCTTCTACGTATGTCCCCGGGATGGCCGGACCCTTTCAGAAGCTAGAAGG
coGALVenv  CTAGAGCCAGAACCAGAATGGCCAGCAGCACCTTCTACGTGTGCCCCAGAGATGGCAGAACCCTGAGCGAGGCTAGAAGA

                  490       500       510       520       530       540       550       560
           ....|....|....|....|....|....|....|....|....|....|....|....|....|....|....|....|
GALVenv    TGCGGGGGGCTAGAATCCCTATACTGTAAAGAATGGGATTGTGAGACCACGGGGACCGGTTATTGGCTATCTAAATCCTC
coGALVenv  TGTGGCGGCCTGGAAAGCCTGTACTGCAAAGAGTGGGACTGCGAGACAACCGGCACAGGCTACTGGCTGAGCAAGAGCAG

                  570       580       590       600       610       620       630       640
           ....|....|....|....|....|....|....|....|....|....|....|....|....|....|....|....|
GALVenv    AAAAGACCTCATAACTGTAAAATGGGACCAAAATAGCGAATGGACTCAAAAATTTCAACAGTGTCACCAGACCGGCTGGT
coGALVenv  CAAGGACCTGATCACCGTGAAGTGGGACCAGAACAGCGAGTGGACCCAGAAATTCCAGCAGTGCCACCAGACAGGCTGGT

                  650       660       670       680       690       700       710       720
           ....|....|....|....|....|....|....|....|....|....|....|....|....|....|....|....|
GALVenv    GTAACCCCCTTAAAATAGATTTCACAGACAAAGGAAAATTATCCAAGGACTGGATAACGGGAAAAACCTGGGGATTAAGA
coGALVenv  GCAACCCTCTGAAGATCGACTTCACCGACAAGGGCAAGCTGTCCAAGGATTGGATCACCGGCAAGACCTGGGGCCTGCGC

                  730       740       750       760       770       780       790       800
           ....|....|....|....|....|....|....|....|....|....|....|....|....|....|....|....|
GALVenv    TTCTATGTGTCTGGACATCCAGGCGTACAGTTCACCATTCGCTTAAAAATCACCAACATGCCAGCTGTGGCAGTAGGTCC
coGALVenv  TTCTATGTGTCTGGACATCCTGGCGTGCAGTTCACCATCCGGCTGAAGATCACCAACATGCCCGCTGTGGCTGTGGGCCC

                  810       820       830       840       850       860       870       880
           ....|....|....|....|....|....|....|....|....|....|....|....|....|....|....|....|
GALVenv    TGACCTCGTCCTTGTGGAACAAGGACCTCCTAGAACGTCCCTCGCTCTCCCACCTCCTCTTCCCCCAAGGGAAGCGCCAC
coGALVenv  TGATCTGGTGCTTGTTGAACAGGGCCCTCCTAGAACCTCTCTGGCTTTGCCTCCTCCACTGCCTCCTAGAGAAGCCCCTC

                  890       900       910       920       930       940       950       960
           ....|....|....|....|....|....|....|....|....|....|....|....|....|....|....|....|
GALVenv    CGCCATCTCTCCCCGACTCTAACTCCACAGCCCTGGCGACTAGTGCACAAACTCCCACGGTGAGAAAAACAATTGTTACC
coGALVenv  CACCTAGCCTGCCTGACAGCAATTCTACAGCCCTGGCCACCAGCGCTCAGACCCCTACAGTGCGGAAAACCATCGTGACC

                  970       980       990      1000      1010      1020      1030      1040
           ....|....|....|....|....|....|....|....|....|....|....|....|....|....|....|....|
GALVenv    CTAAACACTCCGCCTCCCACCACAGGCGACAGACTTTTTGATCTTGTGCAGGGGGCCTTCCTAACCTTAAATGCTACCAA
coGALVenv  CTGAACACCCCTCCTCCAACCACCGGCGACAGACTGTTTGATCTGGTCCAGGGCGCCTTCCTGACACTGAACGCTACAAA

                 1050      1060      1070      1080      1090      1100      1110      1120
           ....|....|....|....|....|....|....|....|....|....|....|....|....|....|....|....|
GALVenv    CCCAGGGGCCACTGAGTCTTGCTGGCTTTGTTTGGCCATGGGCCCCCCTTATTATGAAGCAATAGCCTCATCAGGAGAGG
coGALVenv  TCCTGGCGCCACCGAGAGCTGTTGGCTGTGTCTTGCTATGGGCCCACCTTACTACGAGGCCATTGCCTCTAGCGGCGAGG
```

```
                1130      1140      1150      1160      1170      1180      1190      1200
GALVenv    TCGCCTACTCCACCGACCTTGACCGGTGCCGCTGGGGGACCCAAGGAAAGCTCACCCTCACTGAGGTCTCAGGACACGGG
coGALVenv  TGGCCTATAGCACCGACCTGGATAGATGCAGATGGGGCACCCAGGGAAAGCTGACCCTGACAGAAGTTTCTGGCCACGGC

                1210      1220      1230      1240      1250      1260      1270      1280
GALVenv    TTGTGCATAGGAAAGGTGCCCTTTACCCATCAGCATCTCTGCAATCAGACCCTATCCATCAATTCCTCCGGAGACCATCA
coGALVenv  CTGTGCATCGGCAAGGTGCCCTTTACACACCAGCACCTGTGCAATCAGACCCTGAGCATCAACAGCAGCGGCGACCACCA

                1290      1300      1310      1320      1330      1340      1350      1360
GALVenv    GTATCTGCTCCCCTCCAACCATAGCTGGTGGGCTTGCAGCACTGGCCTCACCCCTTGCCTCTCCACCTCAGTTTTTAATC
coGALVenv  GTACCTGCTGCCTAGCAATCATTCTTGGTGGGCCTGCAGCACCGGCCTGACACCTTGTCTGAGCACCAGCGTGTTCAACC

                1370      1380      1390      1400      1410      1420      1430      1440
GALVenv    AGACTAGAGATTTCTGTATCCAGGTCCAGCTGATTCCTCGCATCTATTACTATCCTGAAGAAGTTTTGTTACAGGCCTAT
coGALVenv  AGACCAGGGACTTCTGCATCCAAGTGCAGCTGATCCCCAGGATCTACTACTACCCCGAGGAAGTGCTGCTCCAGGCCTAC

                1450      1460      1470      1480      1490      1500      1510      1520
GALVenv    GACAATTCTCACCCCAGGACTAAAAGAGAGGCTGTCTCACTTACCCTAGCTGTTTTACTGGGGTTGGGAATCACGGCGGG
coGALVenv  GACAATTCTCACCCTCGGACCAAGCGGGAAGCCGTGTCTCTGACACTGGCTGTGCTGCTCGGACTGGGAATCACAGCCGG

                1530      1540      1550      1560      1570      1580      1590      1600
GALVenv    AATAGGTACTGGTTCAACTGCCTTAATTAAAGGACCTATAGACCTCCAGCAAGGCCTGACAAGCCTCCAGATCGCCATAG
coGALVenv  AATCGGAACAGGCAGCACTGCCCTGATCAAGGGCCCTATCGATCTGCAGCAGGGACTGACCTCTCTGCAGATTGCCATCG

                1610      1620      1630      1640      1650      1660      1670      1680
GALVenv    ATGCTGACCTCCGGGCCCTCCAAGACTCAGTCAGCAAGTTAGAGGACTCACTGACTTCCCTGTCCGAGGTAGTGCTCCAA
coGALVenv  ACGCCGATCTGAGAGCCCTGCAGGATAGCGTGTCCAAGCTGGAAGATAGCCTGACCAGCCTGAGCGAAGTCGTGCTGCAG

                1690      1700      1710      1720      1730      1740      1750      1760
GALVenv    AATAGGAGAGGCCTTGACTTGCTGTTTCTAAAAGAAGGTGGCCTCTGTGCGGCCCTAAAGGAAGAGTGCTGTTTTTACAT
coGALVenv  AATAGAAGAGGCCTGGACCTGCTGTTCCTGAAAGAAGGCGGACTTTGCGCCGCTCTGAAAGAGGAATGCTGTTTCTACAT

                1770      1780      1790      1800      1810      1820      1830      1840
GALVenv    AGACCACTCAGGTGCAGTACGGGACTCCATGAAAAAACTCAAAGAAAAACTGGATAAAAGACAGTTAGAGCGCCAGAAAA
coGALVenv  CGACCACTCTGGCGCCGTGCGGGACAGCATGAAGAAGCTGAAAGAGAAGCTGGACAAGCGCCAGCTGGAAAGACAGAAGT

                1850      1860      1870      1880      1890      1900      1910      1920
GALVenv    GCCAAAACTGGTATGAAGGATGGTTCAATACCTCCCCCTGGTTTACCACCTTAATCTCCACCATCATGGGACCTCTAATA
coGALVenv  CTCAGAATTGGTACGAAGGCTGGTTCAACACAAGCCCCTGGTTCACCACACTGATCAGCACCATCATGGGCCCTCTGATC

                1930      1940      1950      1960      1970      1980      1990      2000
GALVenv    GTACTCTTACTGATCTTACTCTTTGGACCTTGCATTCTCAATCGATTAGTTCAATTTGTTAAAGACAGGATCTCAGTAGT
coGALVenv  GTGCTGCTGCTGATCCTGCTGTTTGGCCCCTGCATCCTGAACAGACTGGTGCAGTTCGTGAAGGACAGAATCAGCGTCGT

                2010      2020      2030      2040      2050      2060
GALVenv    CCAGGCTTTAGTCCTGACTCAACAATACCACCAGCTAAAACCACTAGAGTACGAGCCATGA
coGALVenv  GCAGGCCCTGGTGCTGACACAGCAGTATCACCAGCTGAAGCCTCTGGAATACGAGCCCTGA
```

phCMV-GALVenv-C$_{4070A}$:

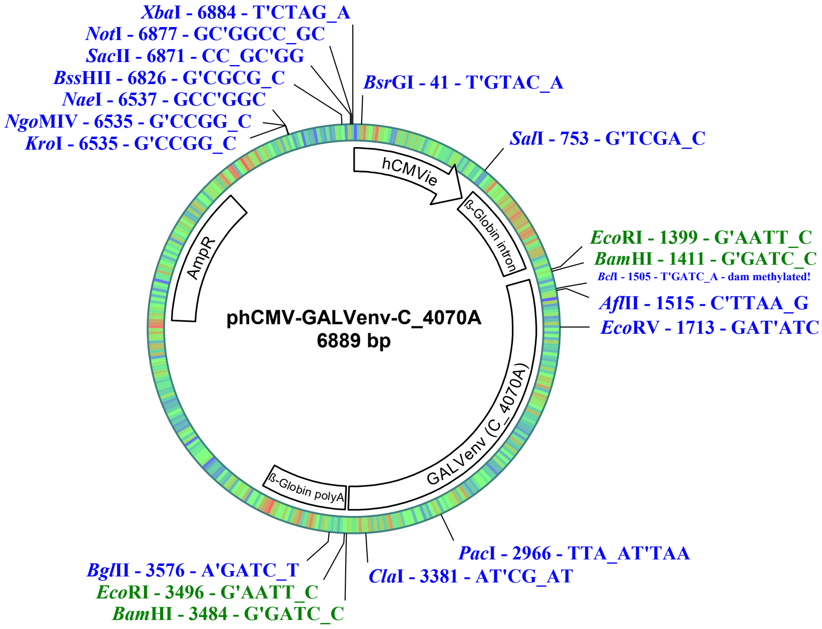


```
                BamHI    NcoI
1401 ATTCCTCGACGGATCCGCCATGGTATTGCTGCCTGGGTCCATGCTTCTCA
                          M   V   L   L   P   G   S   M   L   L   T
```

phCMV-coGALVenv-C$_{4070A}$:

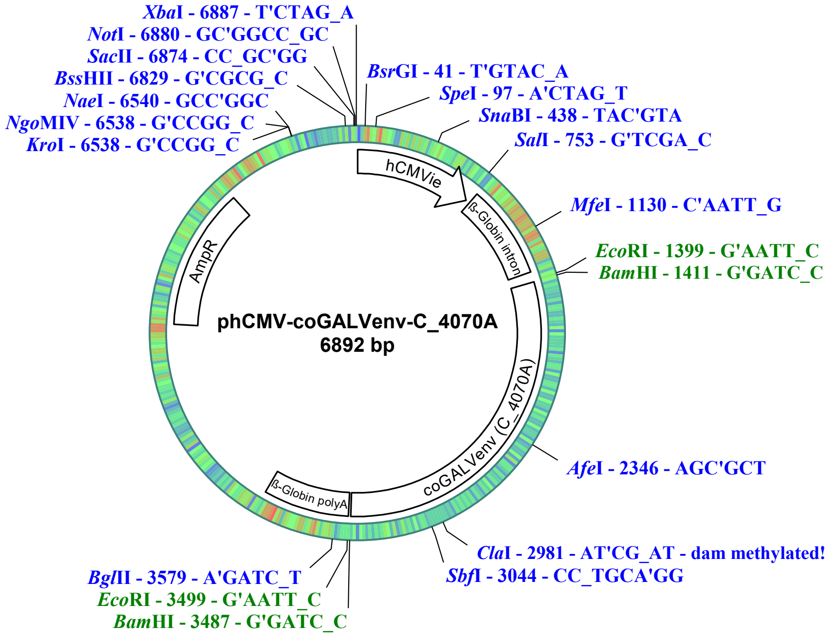


```
                BamHI       NcoI
1401 ATTCCTCGACGGATCCGCCACCATGGTTTTGCTGCCTGGCTCTATGCTGC
                             M   V   L   L   P   G   S   M   L   L
```

Figure S2: Plasmid maps of GALVenv and coGALVenv. Size differs by 3 bp because of shown differences in Kozak sequence (bold). hCMVie: human CMV immediate early promoter; C_4070A: contains C-terminus of amphotropic MLV strain 4070A; AmpR: ampicillin resistance.

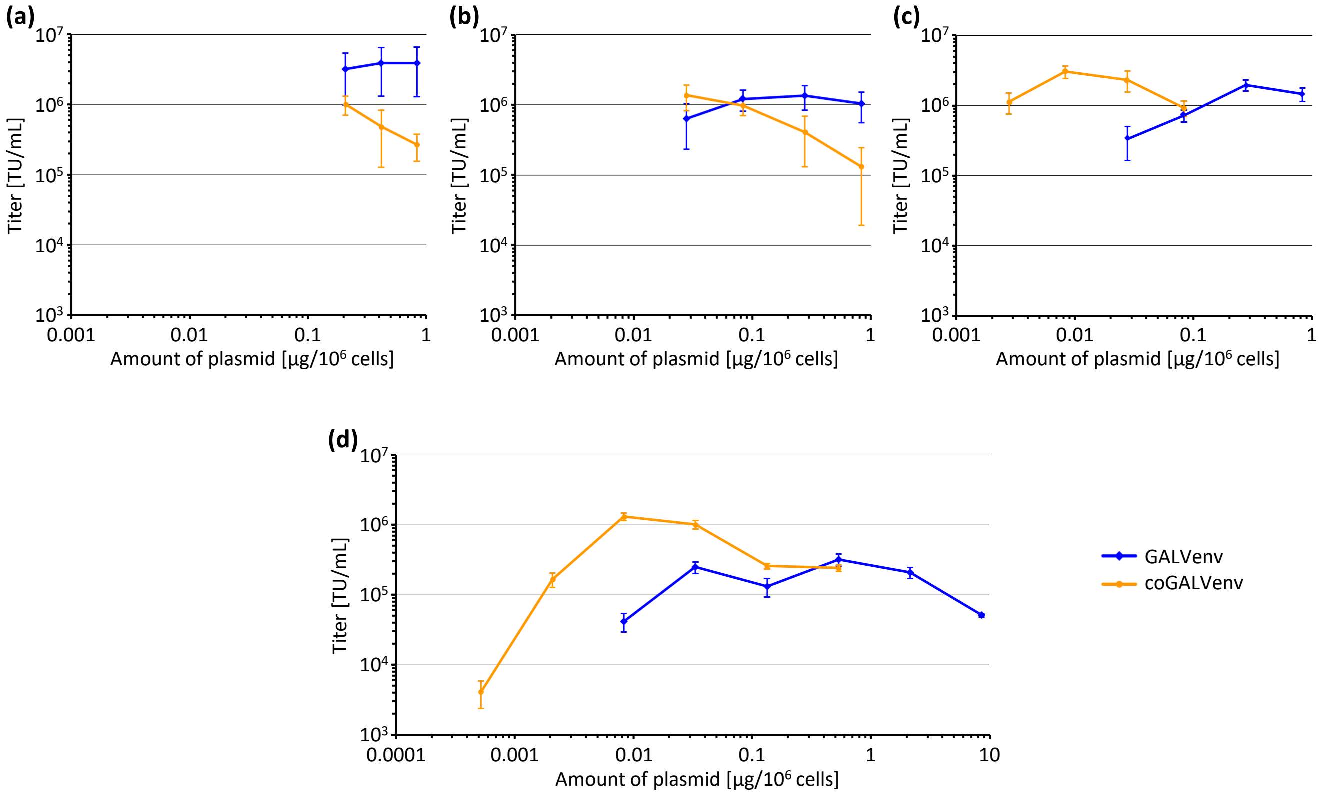

Figure S3: Dose-finding of coGALVenv plasmid amount needed for efficient packaging of lentiviral vectors. **(a)** Starting with conventional amounts of GALVenv and coGALVenv expressing plasmids, the codon-optimized coGALVenv plasmid resulted in lower titers than the non-optimized GALVenv. **(b)** Titer analysis of a larger range of plasmid amounts, the optimum of coGALVenv plasmid amount is still not visible. **(c)** Titer analysis with further reduced amounts of coGALVenv plasmid. The expression strength of the coGALVenv plasmid is more than a log step stronger than the expression strength of the non-optimized GALVenv. **(d)** Vector production at an even further extended range of plasmid amounts, ranging from 0.5 ng to 0.5 µg of coGALVenv expressing plasmid per 10^6 293T producer cells and 8 ng to 8 µg of GALVenv expressing plasmid per 10^6 293T producer cells (data of Fig. S3d also shown as Fig. 1b in main text). For a to d: n = 3
